# Pemphigus Herpetiformis Presenting With Mucosal Involvement: A Rare Clinical Presentation

**DOI:** 10.7759/cureus.106446

**Published:** 2026-04-04

**Authors:** Hawraa Mayouf, Danah Alkandari, Moustafa Hussin, Falah M Alajmi

**Affiliations:** 1 Dermatology, Mubarak Al-Kabeer Hospital, Kuwait City, KWT; 2 Dermatology, Jaber Al-Ahmad Hospital, Kuwait City, KWT; 3 Dermatology, Farwaniya Hospital, Sabah Al-Nasser, KWT

**Keywords:** autoimmune blistering diseases, desmoglein 1, desmoglein-3, direct immunofluorescence, pemphigus herpetiformis, severe mucosal involvement

## Abstract

Pemphigus herpetiformis (PH) is a rare autoimmune blistering disorder, considered a variant of pemphigus, characterized by clinical features resembling dermatitis herpetiformis and immunopathologic findings consistent with pemphigus. Patients typically present with pruritic, urticarial, papular, or vesiculobullous eruptions, while mucosal involvement is uncommon. Due to its overlapping clinical and histopathologic features with those of other blistering diseases, the diagnosis can be challenging.

We report a case of a 49-year-old female who presented with itchy and painful, well-defined erythematous crusted plaques involving the face and upper chest, accompanied by mucosal involvement. Histopathological examination revealed an intraepidermal suprabasal cleft, superficial perivascular inflammatory infiltrate composed of lymphocytes and eosinophils, and intraepidermal vesicles containing neutrophils. Direct immunofluorescence demonstrated intercellular IgG and C3 deposition in a characteristic chicken-wire pattern, consistent with PH. Serologic testing showed strong positivity for desmoglein 1 and desmoglein 3 antibodies. The patient was treated with systemic corticosteroids and mycophenolate mofetil, resulting in an excellent clinical response.

This case highlights the diagnostic challenges and clinical heterogeneity of PH, particularly mucosal involvement, an atypical immunologic profile, and therapeutic response. Increased awareness of this rare entity is essential to ensure accurate diagnosis and appropriate management.

## Introduction

Pemphigus herpetiformis (PH) is a rare and atypical variant within the pemphigus spectrum, characterized by a unique dissociation between clinical features resembling dermatitis herpetiformis and immunopathological findings consistent with pemphigus. It may occur in association with pemphigus foliaceus or pemphigus vulgaris and represents only a small proportion of pemphigus cases worldwide, making it an uncommon and frequently underrecognized entity [1]. PH typically follows a relatively indolent course with fewer complications compared to other pemphigus variants.

Clinically, PH presents with intensely pruritic, erythematous, urticarial, or annular plaques studded with vesicles or pustules, predominantly involving the trunk and proximal extremities [1-3]. Histopathological features typically include eosinophilic or neutrophilic spongiosis with variable acantholysis (loss of intercellular connections between keratinocytes), while direct immunofluorescence demonstrates intercellular IgG deposition in a chicken-wire pattern (net-like intercellular IgG deposition), confirming its classification within the pemphigus group [1-3]. Due to its clinical resemblance to other inflammatory dermatoses, PH is frequently misdiagnosed.

Mucosal involvement in PH is considered uncommon and may complicate differentiation from other pemphigus variants, particularly pemphigus vulgaris [2]. Given its rarity, variable presentation, and diagnostic challenges, reporting additional cases remains important. Herein, we present a rare case of PH with mucosal involvement, highlighting its clinicopathological features.

## Case presentation

A 49-year-old previously healthy female presented with a three-month history of an itchy and painful skin eruption involving the right temple, left forehead, and upper chest. The eruption initially appeared as a vesicular lesion arranged in an annular pattern. She was evaluated at a local polyclinic and was clinically diagnosed with herpes zoster by a family physician, based on the vesicular and annular morphology of the eruption, for which she received oral acyclovir. Partial improvement was noted; however, the persistence of lesions, their progression, and the absence of a dermatomal distribution or associated systemic symptoms made this diagnosis less likely.

The lesions persisted and remained localized to the right temple with extension to the left forehead and upper chest. The patient denied fever, weight loss, or other systemic symptoms. There was no history of similar dermatological conditions, recent infections, or new medication use. Her past medical and surgical history was unremarkable, and she was not taking any regular medications. Family history was non-contributory, and review of systems was otherwise unremarkable. The persistence and progression of the lesions despite antiviral therapy, along with the absence of a dermatomal distribution and lack of systemic symptoms, made the diagnosis of herpes zoster less likely.

**Figure 1:**
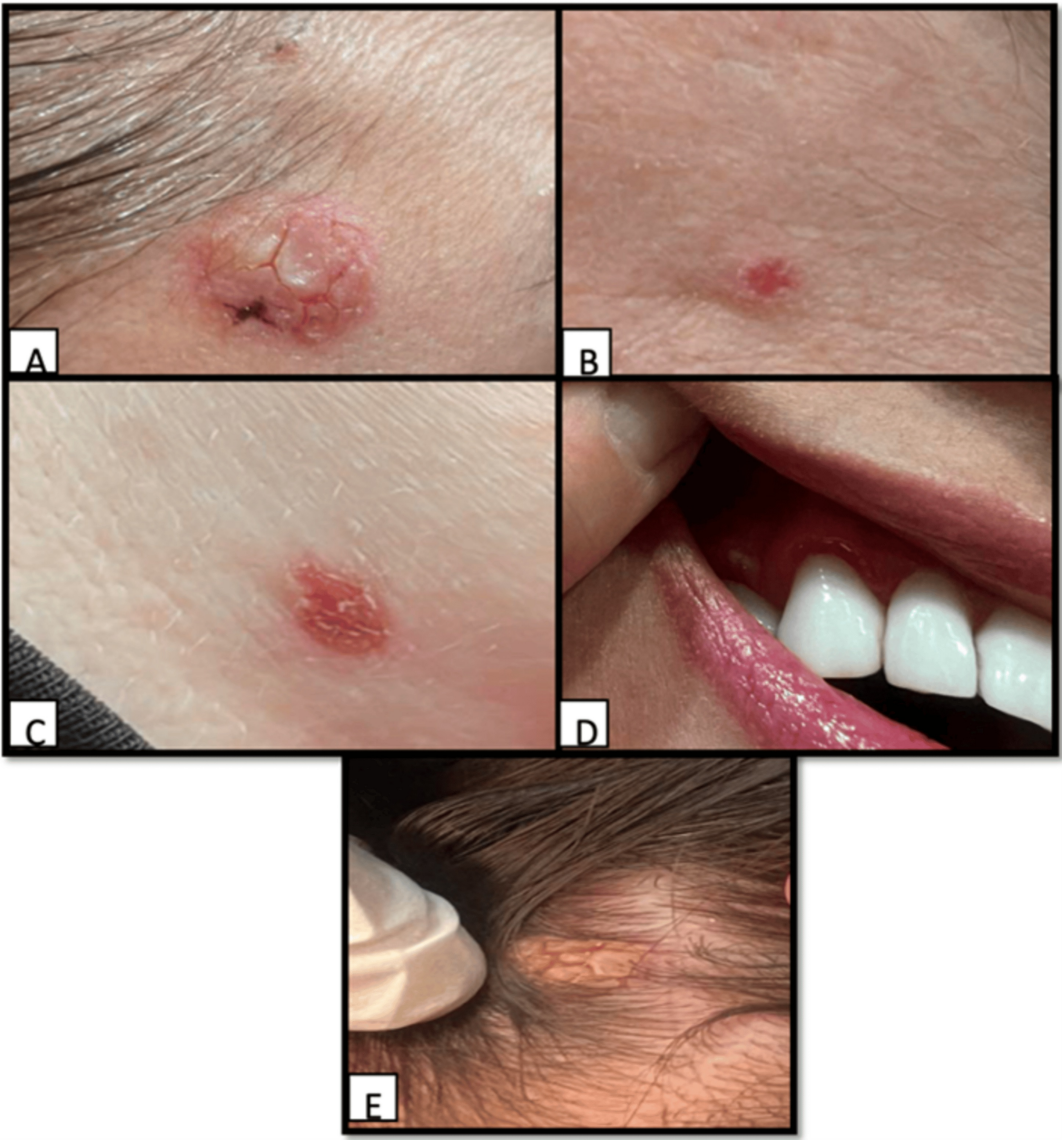
Clinical presentation of cutaneous and mucosal lesions. (A) Erythematous, crusted plaques in an annular arrangement on the right temple. (B) Extension of the eruption to the left forehead. (C) Well-defined erythematous plaques on the upper chest. (D) Gingival inflammation and erosion indicating mucosal involvement. (E) Crusted plaque on the scalp with associated hair involvement.

On physical examination, confluent, well-defined erythematous crusted plaques were observed involving the right temple with extension to the left forehead and upper chest. Examination also revealed gingival inflammation and vaginal erosion, indicating mucosal involvement. In addition, a crusted plaque involving the scalp with associated hair involvement was noted. No nail abnormalities were detected (Figure 1).

**Figure 2:**
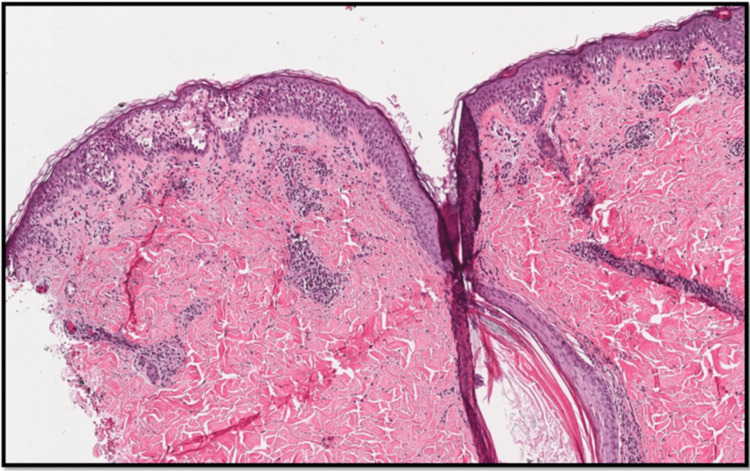
Histopathological findings (H&E stain). A punch biopsy from the right temple demonstrated suprabasal acantholysis resulting in intraepidermal suprabasal cleft, superficial perivascular inflammatory infiltration of lymphocytes and eosinophils, and an intraepidermal vesicle containing neutrophils.

A 4-mm punch biopsy was obtained from the lesion over the right temple for histopathological evaluation. Initial histopathological assessment favored a diagnosis of pemphigus vulgaris, based on the presence of suprabasal intraepidermal clefting and the associated inflammatory infiltrate (Figure 2). Accordingly, the patient was started on oral prednisolone 30 mg daily in combination with mycophenolate mofetil 500 mg twice daily. Significant clinical improvement was noted during follow-up.

**Figure 3:**
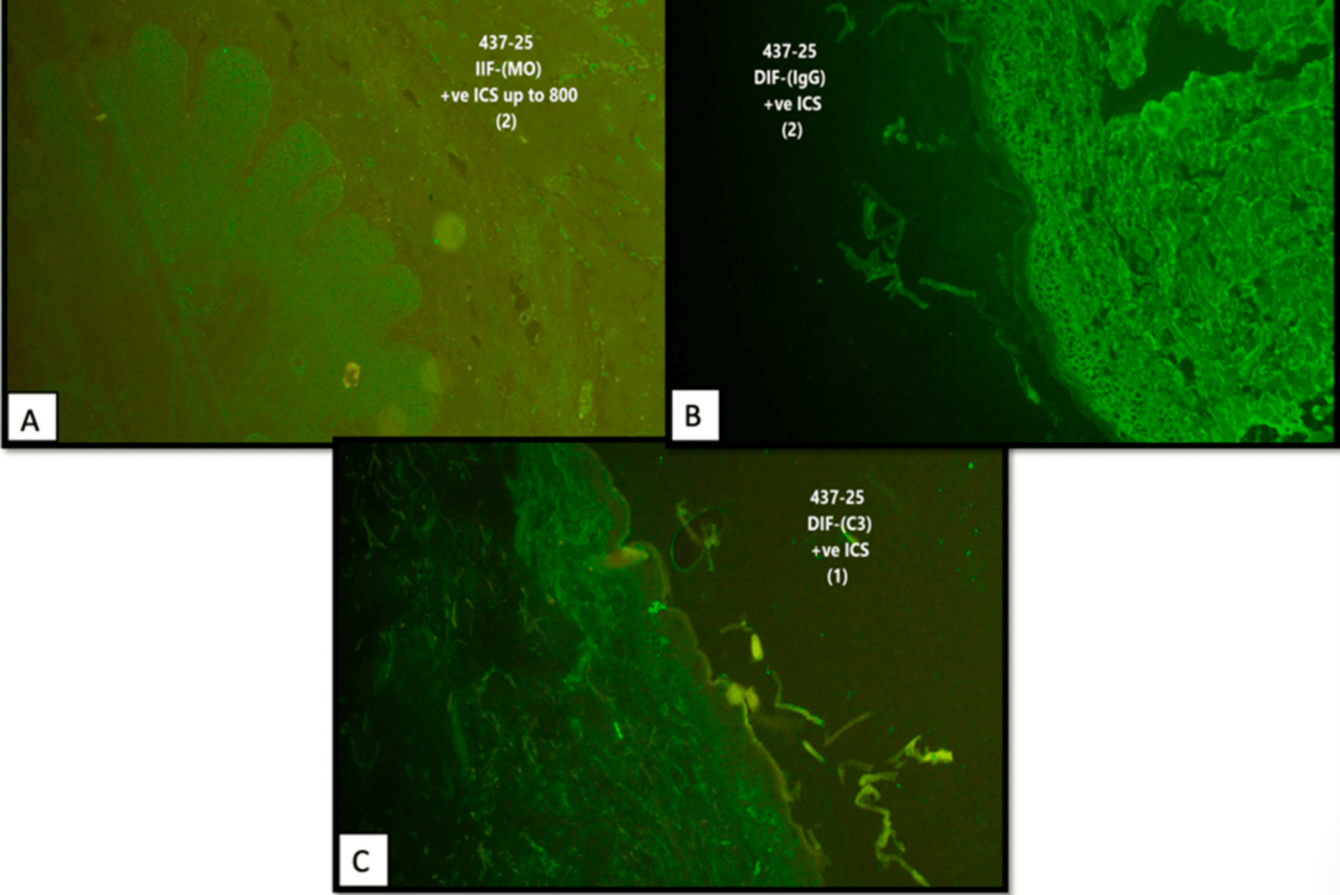
Direct immunofluorescence microscopy. (A, B) Intercellular deposition of IgG and (C) C3 within the epidermis demonstrated a characteristic chicken-wire pattern, confirming the diagnosis of pemphigus herpetiformis.

Subsequent comprehensive clinicopathological review, integrating the clinical morphology, histopathological findings, direct immunofluorescence demonstrating intercellular IgG and C3 deposition in a chicken-wire pattern, and serological testing (enzyme-linked immunosorbent assay (ELISA)), demonstrated elevated levels of both desmoglein 1 (179.416 U/mL) and desmoglein 3 (155.44 U/mL) antibodies, leading to revision of the diagnosis to PH (Figure 3).

Following initiation of therapy, the patient demonstrated significant clinical improvement with resolution of active cutaneous and mucosal lesions. Prednisolone was gradually tapered by 10 mg increments, while mycophenolate mofetil was continued as a steroid-sparing agent. On follow-up, the patient remained clinically stable with sustained disease control and no development of new lesions.

## Discussion

PH is a rare and atypical variant within the pemphigus spectrum, characterized by a dissociation between clinical morphology and immunopathological findings. Clinically, it resembles dermatitis herpetiformis, while immunologically it behaves as pemphigus [1-3]. Most reported cases describe generalized pruritic eruptions predominantly involving the trunk and proximal extremities, with mucosal involvement considered uncommon [1-3].

The present case is notable for several atypical features. First, the disease demonstrated mucosal involvement, including gingival and vaginal erosions. Mucosal involvement in PH has been reported infrequently and is more commonly associated with pemphigus vulgaris [2,3]. Its presence in our patient posed a diagnostic challenge and expanded the differential diagnosis to include other autoimmune bullous disorders. This finding supports the concept that PH exists along a disease spectrum and may share overlapping features with classical pemphigus variants [2].

Second, the disease in our patient showed a localized clinical distribution, involving the right temple, left forehead, upper chest, and scalp, rather than the widespread cutaneous involvement typically described in the literature [1,3]. Localized presentations of PH are rare and may contribute to delayed diagnosis or misdiagnosis, particularly when lesions mimic infectious dermatoses, as occurred in this case, where the patient was initially treated for presumed herpes zoster [1].

Third, serological testing revealed highly positive antibodies to desmoglein 1 and desmoglein 3. PH is most commonly associated with antibodies against desmoglein 1; however, co-positivity for desmoglein 3 has been reported and may correlate with atypical clinical features, including mucosal involvement [1,3]. The presence of both antibodies in our patient may explain the overlap between herpetiform cutaneous morphology and mucosal disease, further highlighting the heterogeneity of the immunological profile in PH [3].

Histopathological examination in our case demonstrated an intraepidermal suprabasal cleft with inflammatory infiltrates composed of lymphocytes, eosinophils, and neutrophils, findings consistent with previously described features of PH [1,3]. Direct immunofluorescence showed intercellular deposition of IgG and C3 in a chicken-wire pattern, confirming the diagnosis. Previous reports have also described unusual or mixed immunofluorescence patterns in PH, further emphasizing the importance of comprehensive clinicopathological correlation in atypical cases [4,5].

This case highlights that mucosal involvement, localized disease, and dual desmoglein positivity do not exclude the diagnosis of PH. Awareness of these atypical features is crucial to avoid misdiagnosis and inappropriate treatment. Reporting such cases contributes to expanding the understanding of the clinical and immunological spectrum of PH and supports maintaining a high index of suspicion when evaluating patients with herpetiform eruptions and pemphigus-like immunopathology [1-5].

## Conclusions

PH is a rare and diagnostically challenging variant within the pemphigus spectrum. This case highlights atypical features, including mucosal involvement, localized disease distribution, and dual desmoglein 1 and desmoglein 3 positivity, which may overlap with pemphigus vulgaris and contribute to initial misdiagnosis. Accurate diagnosis relies on careful clinicopathological correlation and dermatopathological review. Early recognition of such atypical presentations is essential to avoid misdiagnosis and guide appropriate management and treatment.
